# Cognitive processing of Chinese verbal irony: the role of reverse adjacency relations in reaction time and accuracy

**DOI:** 10.3389/fpsyg.2025.1619358

**Published:** 2025-08-26

**Authors:** Mian Huang, Zijian Li, Yu Zheng, Caini Yang

**Affiliations:** ^1^School of Foreign Languages and Literatures, Chongqing Normal University, Chongqing, China; ^2^College of Computer and Information Science, Chongqing Normal University, Chongqing, China

**Keywords:** Chinese verbal irony, reverse adjacency relations, cognitive processing, reaction time, accuracy

## Abstract

Traditional theories of irony, such as Grice’s Standard Pragmatic Model and Sperber and Wilson’s echoic mention theory, inadequately explain culture-specific irony processing in Mandarin. This study, building on Huang’s framework which posits that irony comprehension relies on recognizing gradient reverse adjacency relations within conceptual hierarchies, investigates the cognitive mechanisms underlying the comprehension of Chinese verbal irony by employing a self-paced reading and judgment task via an online platform (Wenjuanxing) with 75 native Mandarin speakers, focusing on the role of reverse adjacency relations in reaction time (RT) and accuracy. Participants were required to evaluate 60 sentences whether they are positive or negative comments. These sentences are divided into three conditions: experimental (irony with reverse adjacency relations), control (irony without such relations), and baseline (non-ironic). Results revealed statistically significant difference across conditions, for experimental ironic sentences (6,012 ms) compared to control (4,901 ms) and baseline sentences (3,987 ms all *p* < 0.001). Accuracy rates followed a similar pattern, with the experimental condition (64.1%) lower than the control (73.6%) and baseline (82.3%, all *p* < 0.01). These findings highlight the cognitive cost of resolving reverse adjacency relations, supporting Huang’s framework, challenging universalist models and emphasizing the need for culture-sensitive frameworks in figurative language studies. Additionally, this study suggests un explored factors influencing irony comprehension in terms of a speed-accuracy tradeoff by a moderate positive correlation (*r* = 0.4, *p* < 0.001) between RT and accuracy cross all conditions and advances cognitive pragmatics by integrating relational hierarchies into irony theories and offers implications for cross-cultural communication, language pedagogy, and NLP systems.

## 1 Introduction

The term “irony,” as a kind of rhetorical devices and humor, it has long been favored by scholars in Chinese. Traditionally, irony is regarded as a figure of speech, and has always been studied in literature and rhetoric (Chen, 2005). For instance, to express that today’s weather is terrible, one might use the ironic rhetorical device and say, “What a nice and warm day today.” Such an ironic approach not only demonstrates the awful weather but also shows the author’s sense of helplessness towards it. By using irony, the author’s psychological activities can be presented more vividly and fully, while also allowing the reader to empathize with the character’s mental state when uttering such words (Hong, 2023).

Recently, with the rise of cognitive linguistics in the 1980s, it has also injected new impetus into the research of verbal irony. It not only regards irony as a device of rhetoric, but also treats the irony from the perspective of cognitive psychology. With the increasing prevalence and deepening of communication in daily life, the cognitive psychology and neurology of irony have attracted more and more attention from scholars, and irony has gradually become a hot subject of psycholinguistics. In recent years, the research mainly focuses on the following four aspects: the cognitive mechanism and understanding process of irony, the definition and connotation of irony, the restriction condition and inducing mechanism of irony production and understanding, and the pragmatic motivation and value of irony (Wang, 2016). Therefore, many western scholars have put forward various theories on irony. We will make an overview about these theories and the methods scholars apply in empirical studies to confirm theories.

Early pragmatic investigations originated from the Standard Pragmatic Model proposed by Grice and Searle, which posits that speakers generally adhere to “cooperative principles” by following established conversational maxims. Violations of these maxims indicate either non-cooperation or intentional conveyance of meanings beyond literal interpretation (Grice, 1975, 1978; Searle, 1978). This model assumes a bottom-up processing mechanism where literal meaning is initially analyzed before contextual compatibility assessment, characterizing irony as fundamentally negative – a linguistic phenomenon manifesting opposition between “what is said” and “what is meant.” However, this framework fails to explain why conventionalized irony demonstrates enhanced processing efficiency. Then, Giora and Fein’s Graded Salience Hypothesis (GSH) provides crucial theoretical advancement. This framework proposes that salient meanings (influenced by lexical frequency, familiarity, conventionality, and prototypicality) receive prior activation over non-salient meanings during irony comprehension. It emphasizes that comprehension of irony extends beyond literal meaning analysis, requiring contextual integration, detection of pragmatic maxim violations, and cognitive schema deviation to infer speakers’ genuine intentions and emotional states (Giora, 1997). Subsequently, Clark and Gerrig conceptualized verbal irony as a “pretense” behavior, in which the speaker pretends to be ignorant, expresses an attitude through literal statements, and implicitly expects the audience to discern ironic or opposing meanings (Clark and Gerrig, 1984). However, this framework fails to fully address the issue of how irony interacts with the audience’s socio-cultural knowledge, fails to clarify the speaker’s attitude, and is difficult to explain non-disguised ironic expressions. Sperber and Wilson’s “Relevance: Communication and Cognition” introduced the theory of echoic mention, which suggests that irony, as echoic speech, reflects the speaker’s evaluative stance towards the audience, shifting the “oppositional meaning” to “echoic” interpretations. By using contemptuous or mocking markers, the speaker maintains a distance from the corresponding proposition, thereby achieving a satirical effect (Sperber and Wilson, 1986). This theory critiques the limitations of pretense-based explanations, arguing that irony constitutes context-driven critical comprehension rather than mere falsehood simulation. The echoic framework lays theoretical groundwork for subsequent models like the Direct Access Model, emphasizing direct inferential processes in irony comprehension. And the Direct Access Model proposed by Gibbs represents a significant theoretical advancement, challenging the traditional sequential processing assumption that prioritizes literal meaning derivation. It believes that rich contextual information enables people to immediately obtain satirical explanations without the need for mandatory textual analysis, indicating that the processing efficiency between satire and textual expression is comparable (Gibbs, 1994).

In summary, these theories and models have different perspectives on the cognitive processing and understanding of irony. But overall, they cannot completely cover all examples of irony. Let us take a look at the following example of irony in Chinese:

(1) Context: 你总是关心别人并照顾他人 (You always care about others and take care of them).

Comment: 你可真是让人讨厌啊 (You are such an annoying person).

Example (1) is a conventional expression in accordance with reality and meets our expectation in daily life. But this seemingly universal answer cannot be answered by the theories mentioned above. For example, Clark’s pretense theory mentions that the speaker is playing the role of an injudicious person, but the response does not indicate this perspective. The echoic account is also different from the answer, as it cannot tell what the speaker is echoing about. The fundamental limitation of existing paradigms is that they mainly focus on semantic oppositions at the surface level (such as literal and intentional meanings), while ignoring the essence of irony as a cognitive language phenomenon. This limitation is further underscored by Pexman’s (2008) parallel-constraint-satisfaction approach, which emphasizes that irony comprehension arises from the simultaneous integration of multiple probabilistic cues, including context, shared knowledge, speaker characteristics, and emotional tone, rather than a sequential analysis of literal meaning followed by pragmatic inference. In view of this, we believe that irony is constrained by the relational knowledge of the knowledge structure in people’s brains, and is the feeling of a certain relationship in a specific environment. Example 1, specifically, regards irony as a grasp of different degrees of reverse adjacent relationships.

At its core, irony represents a specialized form of human intuition that systematically exploits conventional relational frameworks to capture and emphasize deviations within reverse adjacent conceptual subdomains. Irony like Example 1 demonstrates a new theoretical proposition proposed by Huang, which is that “ironic cognition fundamentally resides in the recognition and manipulation of gradient reverse adjacency relations within conceptual hierarchies” (Huang, 2008b), validated by Xu’s Model-Based Pragmatic Reasoning framework (Xu, 2007)—a cognitively grounded approach emphasizing the role of mental model construction in pragmatic inference.

Xu posits that in the pragmatic derivation of meaning during discourse comprehension, conventional relations serve as cognitive foundations for interpreting linguistic expressions. These relations manifest through implicit expressions that systematically elaborate or complement explicit expressions, thereby transforming fragmented utterances into coherent communicative acts via conventionalized inferential processes (Xu, 2003; Xu, 2004; Xu, 2006a; Xu, 2006b). Fundamentally, conventional relations operate as compact knowledge clusters distributed across neural knowledge architectures, forming intricate conceptual networks through adjacent and similarity-based interconnections. This relational framework constitutes a cognitive foundation for organizing experiential knowledge – specifically, entities bearing adjacent/similar relationships exhibit existential implication, where the presence of X intrinsically entails the potential presence of Y within appropriate contextual parameters (Xu, 2006b; Xu, 2007). Building upon this theoretical framework, Huang (2008a, 2008b) elucidates the cognitive mechanics of verbal irony implementation, proposing that ironic communication fundamentally operates through adjacent relations. As in Example (1), the ironic meaning arises through a process of gradient reversal within a conceptual hierarchy of evaluative adjectives. Starting from the literal term “讨厌 (tǎoyàn, annoying),” comprehension involves intuitively traversing adjacent relational nodes in the opposite evaluative direction: first to “不那么讨厌 (bù nàme tǎoyàn, less annoying or not so annoying),” and continuing stepwise until reaching the opposite semantic extreme “喜欢 (xǐhuān, liking).” Crucially, this reversal exploits conventional relational knowledge structures (e.g., the adjacency and opposition within an evaluative scale like dislike ↔ like) embedded in shared cultural cognition. The speaker’s ironic intent (praise disguised as criticism) is thus realized not merely through semantic opposition but through the cognitive manipulation of these gradient reverse adjacency relations. Huang’s theoretical innovation posits that both the production and comprehension of irony constitute an instinctive cognitive process wherein individuals, constrained by conventional knowledge structures within neural architectures, intuitively apprehend different degrees of reverse adjacent relationships in context-specific situations.

Recent research in experimental pragmatics has provided strong theoretical support. Experimental pragmatics studies can reveal the cerebral mechanisms, neural mechanisms, and psychological mechanisms underlying pragmatic processing, thereby elucidating the neurophysiological mechanisms of speech production and comprehension (Li, 2021, p. 38). In the study of irony, most scholars use self-paced reading and rating tasks to study the context and individual differences in irony processing (Weng et al., 2023; Zhang et al., 2022; Wang et al., 2023; Ronderos et al., 2024). Some use eye-tracking technology to record participants’ ocular movements during reading tasks (Olkoniemi et al., 2023; Filik et al., 2017; Barzy et al., 2020). To further investigate the neurophysiological mechanisms underlying irony processing, some researchers have increasingly adopted ERP (event-related potential) techniques, analyzing N400 and P600 components to reveal the cerebral mechanisms involved in irony comprehension (Yang et al., 2020; Pfeifer and Lai, 2021). Beyond these techniques, the field employs diverse experimental paradigms including questionnaire surveys (Huang, 2021), large language model (Liu and Qi, 2024), interview (Kwon et al., 2020) and so forth.

Therefore, based on the above analysis, recent experimental pragmatics research has consistently demonstrated the cognitive cost of irony processing. For instance, Weng et al. (2023) revealed significantly longer reaction times for ironic criticism than literal expressions in Mandarin, with accuracy modulated by relational closeness. Yang et al. (2020) further observed enhanced N400 amplitudes in ERP studies of Chinese irony, indicating heightened semantic integration effort. Cross-linguistic evidence supports this pattern: Filik et al. (2017) documented prolonged gaze durations for English irony using eye-tracking, while Barzy et al. (2020) reported reduced irony comprehension accuracy in autistic adults. These findings collectively establish irony processing as a cognitively demanding task characterized by delayed reaction times (RT) and accuracy tradeoffs—providing critical empirical grounding for our investigation of reverse adjacency relations as a potential cognitive load source. This article explores Huang’s cognitive mechanisms of understanding irony through an online platform “Wenjuanxing” by using self-paced reading task and judgement task. Specifically, by setting up 60 sentences to test college students, we hypothesize that reverse adjacency relations would modulate irony processing efficiency. Following Huang’s (2008b) framework predicting intuitive relational manipulation, we initially anticipated facilitated processing (shorter RTs and higher accuracy) for irony with such relations. However, we also considered the alternative possibility that resolving relational conflicts might impose cognitive costs, potentially prolonging RTs. Meanwhile, our goal was to further analyze whether there was a significant difference in accuracy between these two groups in irony comprehension through paired t-tests. Before conducting the paired t-tests, the following statistical hypotheses were proposed:

Null hypothesis (H0): There is no significant difference in accuracy between the experimental group and the control group, that is, the mean accuracy of the two groups is equal.

Alternative hypothesis (H1): There is a significant difference in the accuracy between the experimental group and the control group, that is, the mean accuracy of the two groups is not equal.

Critically, this study makes three key contributions: (1) It provides the first empirical validation of Huang’s (2008b) reverse adjacency theory, addressing a theoretical gap in culture-specific irony processing; (2) It challenges universalist models (e.g., Gricean and echoic frameworks) by demonstrating how Mandarin irony relies on relational hierarchies rather than semantic opposition alone; (3) It offers practical insights for cross-cultural communication training and NLP systems handling Chinese figurative language, where conventional irony detection algorithms often fail.

## 2 Materials and methods

### 2.1 Participants

Seventy-five participants (30 males, 45 females; mean age = 24.13, range = 22–26) were recruited and compensated for this experiment. Participants were solicited online, including students from Chongqing Normal University and other institutions, as well as some workers with the same range age. All participants were native Mandarin speakers, right-handed, with normal or corrected-to-normal vision, and no history of speech/hearing impairments or neurological/psychiatric disorders. Written informed consent was obtained prior to the experiment. This study was approved by the Institutional Review Board of Chongqing Normal University (Approval No. CNU-IRB2023-0098), ensuring compliance with ethical standards for human participants’ research. No participants reported awareness of the experimental purpose during the procedure, and no data were excluded due to artifacts.

To ensure basic linguistic comparability across participants, although participants were not formally assessed for reading habits, all had attained at least a bachelor’s degree or equivalent, ensuring a comparable level of literacy and academic exposure necessary for processing written Mandarin. Given their educational backgrounds, all participants were presumed to possess sufficient proficiency to understand and interpret written materials, including figurative language such as irony.

Although the current study did not focus on individual difference variables, potential variation in linguistic background (e.g., language use frequency, exposure to irony in workplace vs. academic discourse) is acknowledged as a possible confounding factor. This limitation is further discussed in Section 4.

### 2.2 Materials

The experiment employed self-designed sentence materials, comprising 60 comment sentences and 60 corresponding contextual sentences, evenly divided into three groups: experimental, control, and baseline. Each sentence contained 10 characters, while each context contained 12 characters (see Table 1 for examples).

**Table 1:** Experimental stimuli example.

Group	Example Stimuli	
	Context	Comment Sentence
Irony with reverse adjacent relations	你面对突发事件半天才反应.	你最近反应速度太快了.
	ni miandui tufashijian Bantian caifanying.	ni zuijin fanyingsudu taikuaile
	you take a long time to react to unexpected events.	You’ve been reacting too quickly lately.
Irony without reverse adjacent relations	某个晚上突然心绞痛又犯了.	我真是人品大爆发了么.
	mouge wanshang turan xinjiaotong fanle.	wo zhenshi renpin dabaofa leme.
	you suddenly had angina again one night.	have I really had a great outburst of luck.
Declarative sentence without irony	他总乐于助人从不推辞别人.	他真是个乐于助人的人.
	ta zongleyuzhuren congbu tuici bieren.	ta zhenshige leyuzhuren de ren.
	he is always willing to help others and never rejects them.	he is truly a helpful person.

The experimental group included 20 ironic sentences featuring inverse adjacency relations. The control group contained 20 ironic sentences without inverse adjacency relations. The baseline group consisted of 20 non-ironic declarative sentences. Each sentence was paired with a contextual sentence to ensure semantic coherence during the experiment.

The baseline condition, consisting of literal non-ironic sentences, was included as a baseline to control for cognitive and linguistic factors such as sentence length, syntactic complexity, and general reading time. Its purpose was to provide a benchmark against which the additional cognitive cost of irony processing could be assessed.

Participants were not asked to make any binary judgments (e.g., “Ironic” vs. “Not Ironic”) for these sentences; thus, no accuracy data were collected for this condition. It was included solely for comparison of reaction times, not for response-based correctness analysis. This design choice ensures that any observed RT differences between ironic and non-ironic sentences are attributable to the pragmatic demands of irony comprehension, rather than basic linguistic processing differences.

Following each sentence, two evaluation options were provided: [positive comment] and [negative comment], to record participants’ semantic comprehension and attitudinal responses.

To familiarize participants with the experimental procedure, five practice sentences—all containing ironic expressions with reverse adjacent relations—were administered prior to the formal experiment. The practice sentences followed the same structural design as the experimental trials but were excluded from data analysis.

In the formal experiment, all sentences were presented using a self-paced reading paradigm, with participants’ reaction times and evaluation choices recorded for each sentence set. The time of reading were measured independently for each stimulus to ensure data accuracy and comparability. The order of presentation of materials was randomized to mitigate potential order effects.

Additionally, participants’ accuracy in sentence evaluations was recorded. Accuracy was calculated based on whether their selected evaluations aligned with the experimentally intended outcomes. By analyzing both time of reaction and accuracy rates, a comprehensive assessment of participants’ abilities of comprehension of irony was achieved.

The experimental design aims to investigate the impact of reverse adjacent relations in irony comprehension by comparing reading times and accuracy rates across the three sentence groups. For example, the reverse adjacency relationship in the example in Table 1 exists in the adverbial “too quickly” in the comment sentence. According to Huang’s model, the adverbial “too quickly” can be reversed back to “quickly,” “normal,” “slow,” “too slow.” The reverse process has many stages, and for ease of understanding, we have roughly summarized several ironic grades here. The other experimental sentences are also the same, reverse adjacency relation appears in adjectives, adverbs, and verbs.

While our current three-group design presents a categorical surface structure, it serves as a practical operationalization of this underlying gradient theoretical construct. The experimental irony sentences were carefully constructed to include lexical items—such as adjectives, adverbs, or verbs—that strongly cue scalar reversal, whereas the control irony sentences contain ironic intent without such explicit scalar triggers. In this way, we approximate different degrees of cognitive effort required for conceptual reversal, even though a full parametric continuum is not implemented. Thus, the design remains grounded in the theoretical notion of gradient reverse adjacency, while balancing experimental feasibility.

All materials were validated through pre-testing to ensure semantic clarity and adherence to experimental objectives. To ensure the effectiveness of the experimental materials, we conducted a preliminary experiment before the formal experiment. We recruited 20 participants who did not participate in the formal experiment and their native language is Mandarin (whose demographic characteristics such as age, educational background, etc. are similar to those of the formal experimental participants).

In the pre-experiment, the task of the participants is to: (1) read each combination of context and comment sentence; (2) Determine whether the comment sentence is an irony (yes/no); (3) For sentences judged as ironic, evaluate the comprehensibility of their ironic intent (e.g., using a 5-point or 7-point scale, where 1 = very incomprehensible and 5/7 = very understandable); (4) Choose whether the attitude expressed by the target sentence is positive or negative.

Screening criteria: (a) For ironic sentences in the experimental and control groups, at least 80% of the participants are required to correctly identify them as irony; (b) The average score of irony in the experimental and control groups in terms of ironic comprehensibility needs to be significantly higher than the midpoint of the scale, and there was no significant difference in comprehensibility between the two groups; (c) The accuracy of attitude judgment for all sentences (experimental group, control group, baseline group) needs to reach 90%; (d) The proportion of sentences in the baseline group judged as irony should be less than 5%. According to these standards, sentences that do not meet the requirements have been modified or replaced. The final 60 sets of materials used all meet the above validation standards.

### 2.3 Procedure

The experiment was conducted via the online platform “Wenjuanxing.” Upon accessing the experimental page, participants first provided basic information such as name and gender, followed by instructions detailing the estimated duration, procedures, and a vaguely stated experimental purpose.

Participants received the following written instructions: “In this experiment, you will read a series of sentences and their brief background. Please read each page at your own natural reading speed. Your task is to understand the meaning of the sentence and determine whether the comment attitude expressed by the sentence is positive or negative. After reading each page, the system will ask you to choose one of the two options, [positive comment] and [negative comment], which you think best reflects the attitude of the sentence. Please make a judgment based on your understanding of the meaning of the sentence. The experiment does not require speed, but please focus your attention.”

To familiarize participants with the process, they completed five practice sentences identical in structure to the formal experimental items but excluded from data analysis. The practice phase follows the same process as the formal experimental phase, where participants do not receive any feedback on the correctness of the exercise sentence selection. The purpose of the exercise is only to familiarize participants with the reading interface and selection process. The formal experiment consisted of three pages, each presenting one set of sentences (20 sentences per group: experimental, control, and baseline). The presentation order of sentence groups was randomized.

Participants need to first read each sentence in experimental group sequentially and selected either positive comment or negative comment. After finishing the first page, participants need to click on the next page to read the control group, then the baseline group. Reading time per page was recorded in milliseconds. The system automatically logged participants’ total reading time of the entire page and response choices, calculating accuracy based on predetermined correct answers.

Upon completing all three sentence sets, the experiment terminated automatically, with data saved to the “Wenjuanxing” platform for subsequent analysis. This design aimed to comprehensively analyze reading times and accuracy rates to investigate how reverse adjacent relations influence irony comprehension, while strictly adhering to randomization principles to ensure data reliability and replicability.

### 2.4 Estimation of sample size

To ensure sufficient statistical power for detecting the effects of inverse adjacency relations in Mandarin irony comprehension, an *a priori* power analysis was conducted to determine the minimum required sample size. The analysis, performed using G*Power software, was based on a repeated-measures ANOVA (within-subjects factors).

Parameter selection followed conventions in psychological and linguistic research alongside experimental design specifications: Effect size (f) = 0.25 (medium effect per Cohen’s 1988 criteria, reflecting expected differences in reaction times and accuracy between experimental and control irony conditions). *α* error probability = 0.05 (5% Type I error rate, aligned with disciplinary norms). Power (1 – *β*) = 0.95 (95% probability of detecting true effects; *β* = 0.05). Number of measurements = 3 (experimental irony, control irony, and baseline statements). Number of groups = 1 (within-subjects design with all participants exposed to all conditions). Correlation among repeated measures = 0.5 (moderate correlation assumed). Nonsphericity correction (*ε*) = 1 (sphericity assumed; adjustments to be applied *post hoc* if violated). Input parameters are summarized in Table 2.

**Table 2:** Input parameters for *a priori* power analysis in G*Power.

*f*	α	Power	Groups	Measurements	Corr	ε
0.25	0.05	0.95	1	3	0.5	1

f = effect size; α = Type I error probability; Corr = correlation among repeated measures; ε = nonsphericity correction.

**Figure 1:**
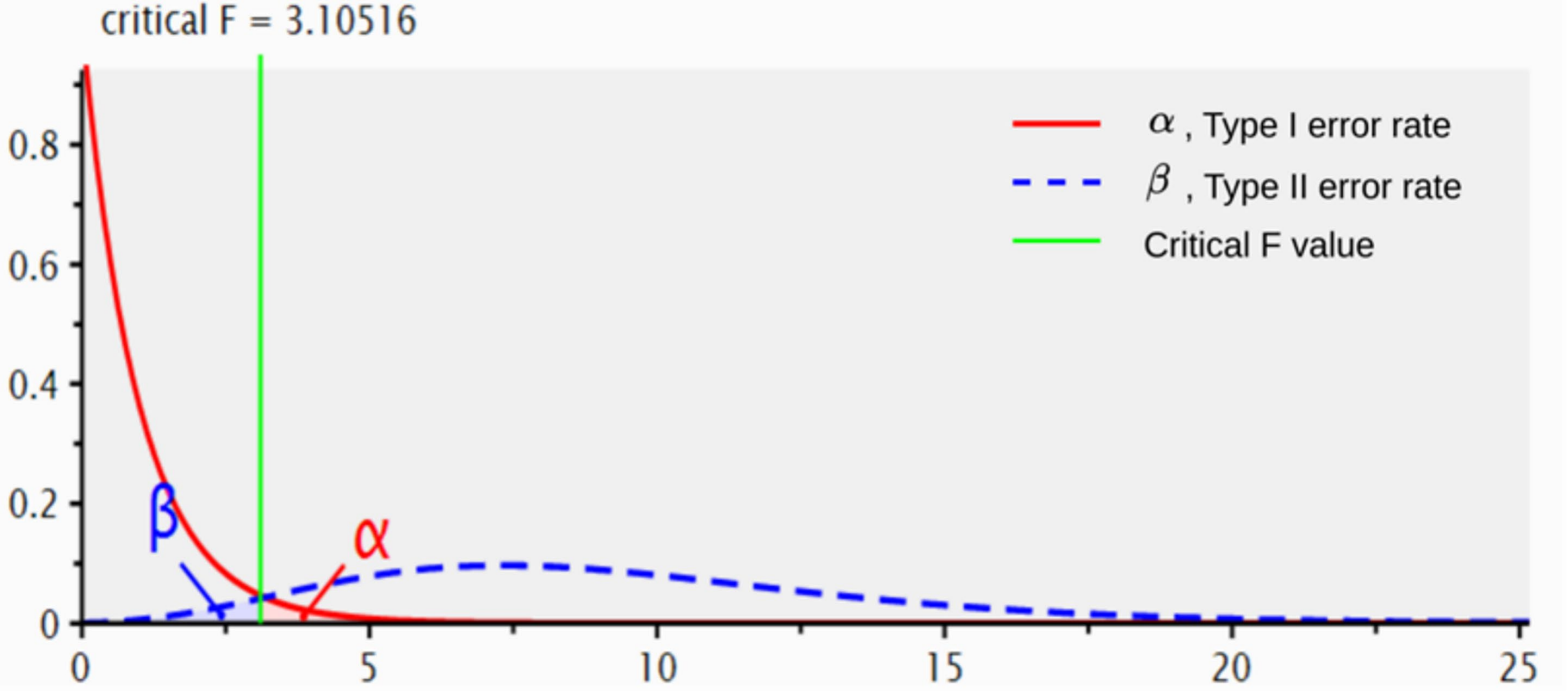
Power curve for repeated-measures ANOVA based on G*Power estimation. The figure illustrates the relationship between *F*-values and error probabilities. The red curve shows Type I error (*α*), the blue curve represents Type II error (*β*), and the green vertical line indicates the critical *F*-value (3.105). Values above this line suggest significant between-condition differences.

Based on the aforementioned parameters, the output from G*Power software provides a detailed analysis of the results, as shown in Table 3 and Figure 1.

**Table 3:** G*Power output summary for sample size estimation.

noncentrality Parameter λ	Critical *F*	Numerator df	Denominator df	Total sample size	Actual power
16.125	3.105	2.000	84.000	43	0.951

The table shows the G*Power output for a repeated-measures ANOVA with three within-subject conditions. λ = noncentrality parameter; Critical F = minimum F value for significance at α = 0.05; df = degrees of freedom; Total sample size = minimum required participants to achieve 95% power.

These results indicate that a minimum sample size of 43 participants is required to achieve 95% statistical power (actual power slightly higher at 0.9514) under the conditions of effect size *f* = 0.25 and significance level *α* = 0.05. This sample size estimation ensures sufficient statistical sensitivity to reliably detect hypothesized effects.

In Figure 1, the x-axis represents *F*-values. As the *F*-value increases, the probability of Type I error (α, red curve) gradually decreases, while the probability of Type II error (β, blue curve) stabilizes. Larger *F*-values indicate more significant between-group differences. The green vertical line marks the critical *F*-value (3.10516 in this figure). If the calculated *F*-value exceeds this critical threshold, the null hypothesis can be rejected, confirming significant differences.

The initial study planned to recruit 75 participants, exceeding the minimum requirement of 43. This provides buffer space for potential data cleaning or participant attrition while enhancing statistical power and result robustness. This analysis treated participants’ reaction times (RT) across three types of sentences (experimental irony, control irony, baseline sentences) as dependent variables: RT_Ex, RT_Ctrl, RT_Refer (unit: ms), with a repeated-measures ANOVA. All 75 valid participants were included, and data were analyzed by using SPSS.

### 2.5 Statistical analysis plan

The statistical analyses focused on two primary dependent variables: reaction time (RT) and comprehension accuracy (ACC). The main independent variable was sentence type, comprising three conditions: (1) ironic sentences with reverse adjacency relations (experimental group), (2) ironic sentences without such structures (control group), and (3) non-ironic literal sentences (baseline group). Demographic variables such as gender and occupational status (student vs. working adult) were also considered in exploratory analyses.

Prior to analysis, RT data were screened for outliers (values > 3 SD from the mean) and averaged per participant within each condition. No transformation was applied, as the distribution of RTs was deemed acceptable for ANOVA given its robustness under conditions of variance homogeneity and adequate sample size. ACC was computed as the proportion of correct binary judgments within ironic conditions. Accuracy was not applicable for the baseline group, as no judgment was required.

Inferential statistics included repeated-measures ANOVA (with Greenhouse–Geisser correction when sphericity was violated), independent samples t-tests for between-group comparisons (gender, occupation), and Pearson correlation analysis to examine the speed-accuracy relationship. Normality was assessed using Shapiro–Wilk tests, and homogeneity of variances via Levene’s test. For the correlation analysis in Section 3.3, only one correlation (RT_Avg vs. ACC_Avg) was computed; thus, no multiple comparison correction was needed.

Exploratory analyses revealed no significant RT differences by gender or background. ACC was slightly higher among female participants and graduate students, but these variables were not included as covariates, as they were not central to our theoretical focus. Their potential role is noted in the Discussion.

### 2.6 ANOVA assumption checks

Before conducting the repeated-measures ANOVA, we tested the key assumptions:

Normality: Shapiro–Wilk tests indicated that the distributions of reaction time and accuracy scores deviated from normality (*p* < 0.05). Even so, prior research has shown that ANOVA is generally robust to such deviations, particularly when sample sizes are reasonable and variances are equal (Schmider et al., 2010; Blanca et al., 2017). Given that the assumption of homogeneity of variance was met, we proceeded with the parametric analysis.

Homogeneity of variance: Levene’s test showed no significant differences in variances across groups (RT: *p* = 0.31; ACC: *p* = 0.21), indicating that the assumption of homogeneity was satisfied.

Sphericity: Mauchly’s test results are reported in Section 3.2.2. Where sphericity was violated, the Greenhouse–Geisser correction was applied.

These assumption checks support the appropriateness of the parametric tests used.

## 3 Results

### 3.1 Sampling heterogeneity analysis

The participant pool consisted of 75 native Mandarin speakers (45 females, 30 males), aged between 20 and 30 (*M* = 24.13, SD = 1.27), including both graduate students (*n* = 62) and undergraduate students (*n* = 13). All participants reported normal or corrected-to-normal vision, and no history of cognitive or linguistic disorders. Educational background was recorded, and all participants had at least a university-level education.

Although the sample was not balanced in terms of gender or educational background, these variables were not central to our theoretical focus. Nevertheless, to assess whether demographic factors influenced experimental outcomes, we conducted exploratory analyses. Independent samples t-tests revealed no significant gender or background differences in reaction time (RT) (gender: *t*(73) = 0.73, *p* = 0.47; background: *t*(73) = 0.57, *p* = 0.58). However, significant differences emerged in accuracy (ACC): female participants performed better than male participants [*t*(73) = 2.52, *p* = 0.014], and graduate students outperformed undergraduates [*t*(73) = 3.84, *p* = 0.001].

These findings indicate that while response speed was not affected by demographic heterogeneity, accuracy may be modulated by gender and educational experience. One possible explanation is that female or more academically advanced participants may possess greater metalinguistic awareness or reading strategies that benefit pragmatic inference. Although such factors were not explicitly modeled in our current study, we now recognize them as potentially meaningful confounds and recommend that future research examine their influence more systematically.

### 3.2 Reaction time differences

Preprocessing of RT Data Prior to Analysis:

For each participant, RT values were averaged within each sentence type to obtain mean RT values across the three conditions.

**Figure 2:**
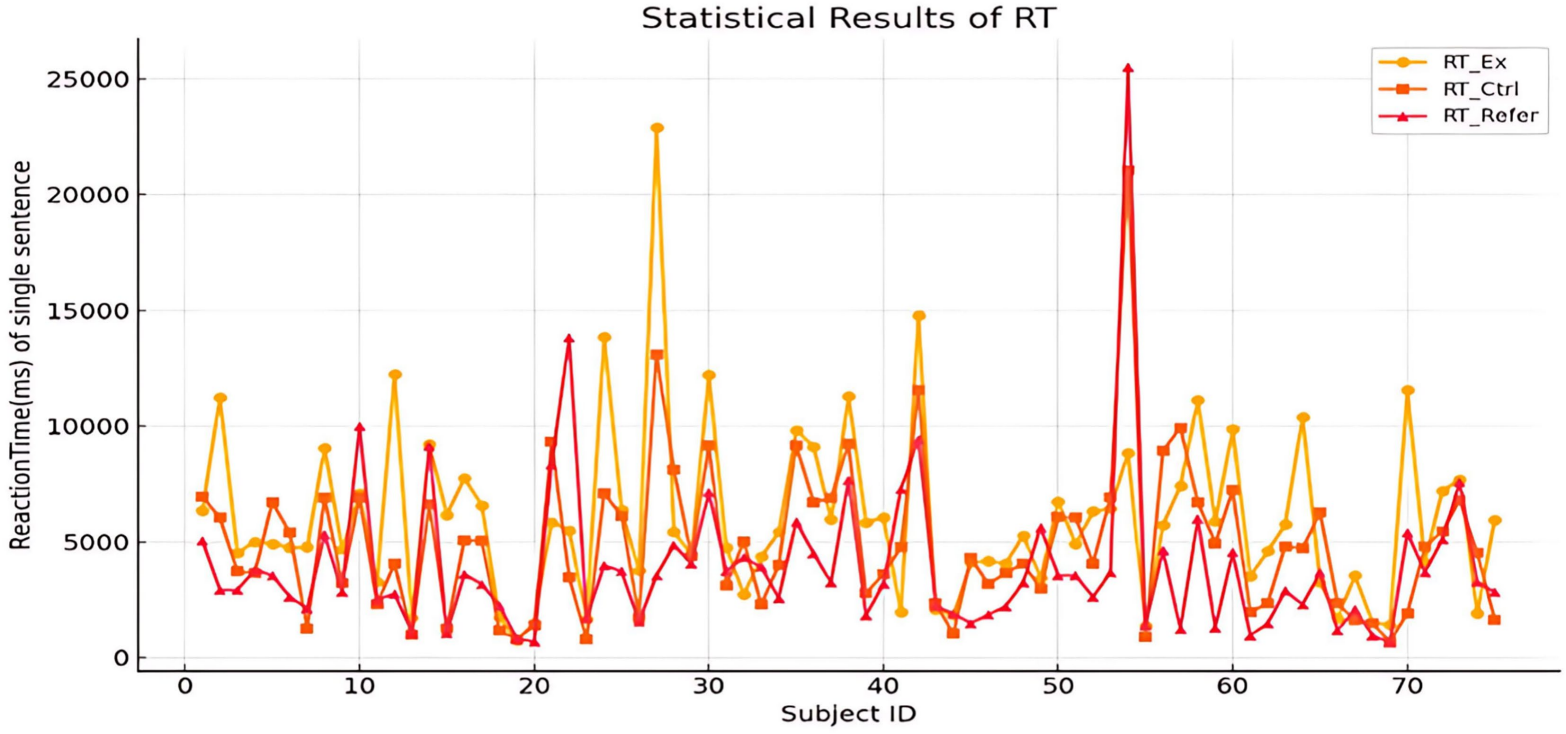
Statistical results of reaction times for individual sentences across conditions. Mean reaction times (ms) by participants (x-axis: Subject ID) across three conditions: RT_Ex (yellow, circles), RT_Ctrl (orange, squares), and RT_Refer (red, triangles).

A line chart (Figure 2) illustrates the mean reaction times of the 75 participants under the three sentence types.

#### 3.2.1 Descriptive statistical analysis

Table 4 displays the mean reaction times and standard deviations across sentence-type conditions.

**Table 4:** Descriptive statistics of reaction times across sentence types.

RT type	Mean	Std	Sample size
RT_Ex (ms)	6012.21	3761.08	75
RT_Ctrl (ms)	4900.81	3342.35	75
RT_Refer (ms)	3987.49	3508.07	75

RT_Ex = Reaction time for experimental ironic sentences; RT_Ctrl = Reaction time for control ironic sentences; RT_Refer = Reaction time for literal baseline sentences. Std = standard deviation.

From Table 4, it can be seen that the reaction time of irony in the experimental group was the longest, followed by the control group, and the reaction time of the referential group was significantly lower overall. All values are in milliseconds. N = 75.

#### 3.2.2 Sphericity test (Mauchly’s test)

To validate the assumptions of repeated-measures ANOVA, Mauchly’s test was conducted. Results indicated violation of sphericity for the sentence-type variable:

Mauchly’s W = 0.664, χ^2^(2) = 29.913, *p* < 0.001 (3.19 × 10^(−7)).

**Figure 3:**
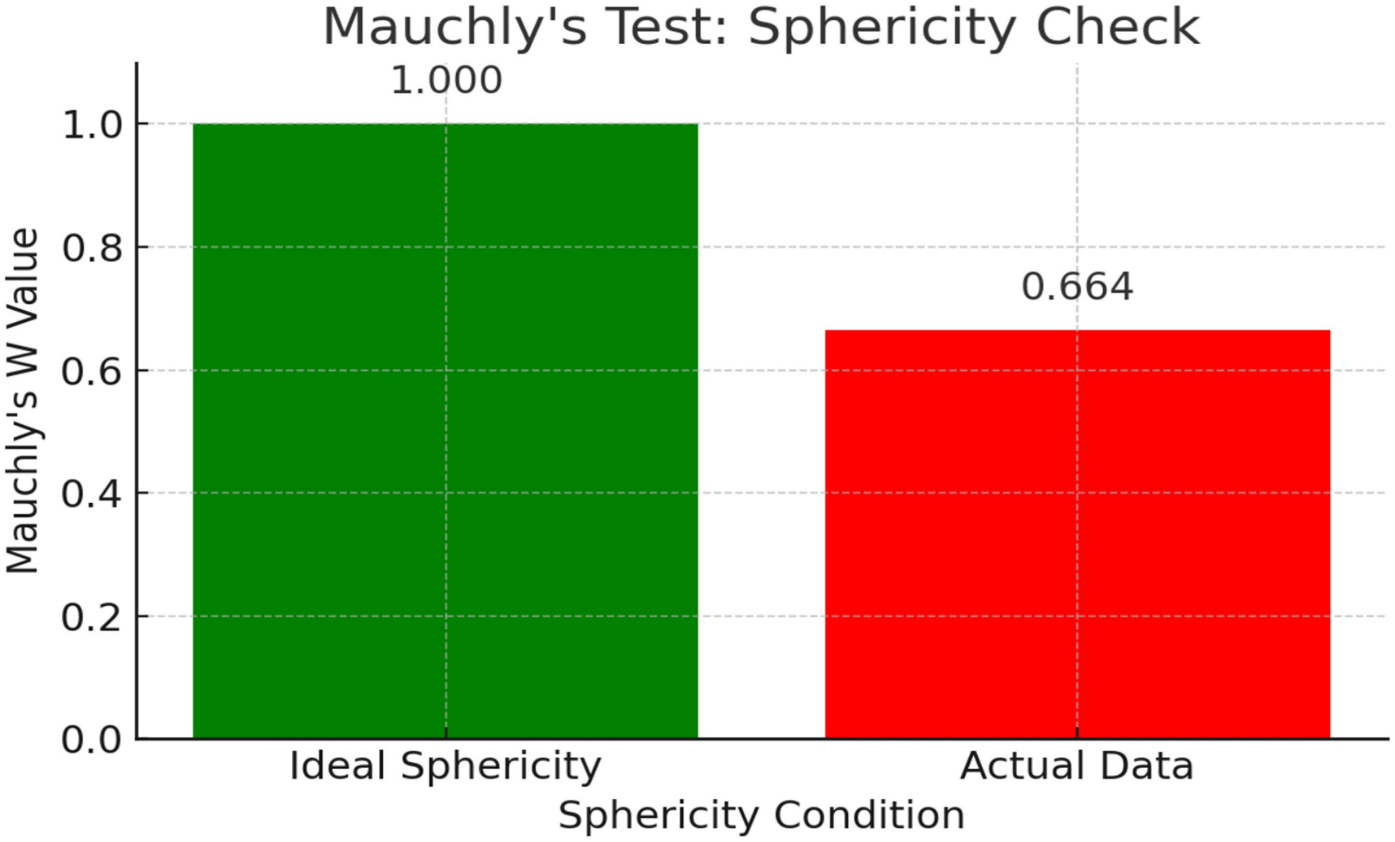
Comparison of ideal and actual sphericity values in Mauchly’s test. The figure illustrates the result of Mauchly’s test of sphericity. The ideal value (*W* = 1.000) represents a perfect sphericity condition, while the actual data (*W* = 0.664) indicates a notable deviation. This significant violation of the sphericity assumption (χ^2^(2) = 29.913, *p* < 0.001) justifies the application of Greenhouse–Geisser correction in the subsequent ANOVA.

Mauchly’s W statistic quantifies sphericity deviation (W = 0.664 suggests notable departure from ideal sphericity, as visualized in Figure 3).

The significant result (*p* < 0.05) confirms heterogeneity of variances across sentence types. Consequently, Greenhouse–Geisser correction was applied to adjust degrees of freedom in subsequent ANOVA to ensure accuracy.

#### 3.2.3 Results of repeated-measures ANOVA

After Greenhouse–Geisser correction, a significant main effect of sentence type on reaction times was observed:

*F*(1.497, 110.704) = 13.852, p < 0.001, η^2^ₚ = 0.158.

This result demonstrates that types of sentences (experimental irony, control irony, baseline sentence) significantly influenced reaction times.

#### 3.2.4 *Post hoc* comparisons (pairwise analyses)

To clarify specific differences between sentence types, pairwise comparisons were conducted. Detailed results are as follows (Table 5):

**Table 5:** Pairwise comparisons of reaction times across sentence types.

Pairwise comparison	Difference mean	Std	*p*	95% confidence interval
RT_Ex vs. RT_Ctrl	1111.397 ms	352.155 ms	0.007	[248.752, 1974.042]
RT_Ex vs. RT_Refer	2024.717 ms	481.817 ms	7.3*10^(−5)	[844.450, 3204.984]
RT_Ctrl vs. RT_Refer	−913.320 ms	298.654 ms	0.009	[−1644.909, −181.731]

RT_Ex = Reaction time for experimental irony sentences; RT_Ctrl = Reaction time for control irony sentences; RT_Refer = Reaction time for baseline literal sentences.

“Difference Mean” indicates the mean RT difference between the two conditions compared. Std is standard deviation of the difference. *p* values are Bonferroni-adjusted. Confidence intervals reflect the 95% range for the difference in mean RTs.

Significant RT differences were observed between experimental irony (RT_Ex) and both control irony (RT_Ctrl) and baseline sentences (RT_Refer). Crucially, the largest disparity occurred between experimental irony and control irony (p < 0.001), while the difference between control irony and baseline sentences was less pronounced (*p* = 0.009).

### 3.3 Accuracy differences

**Figure 4:**
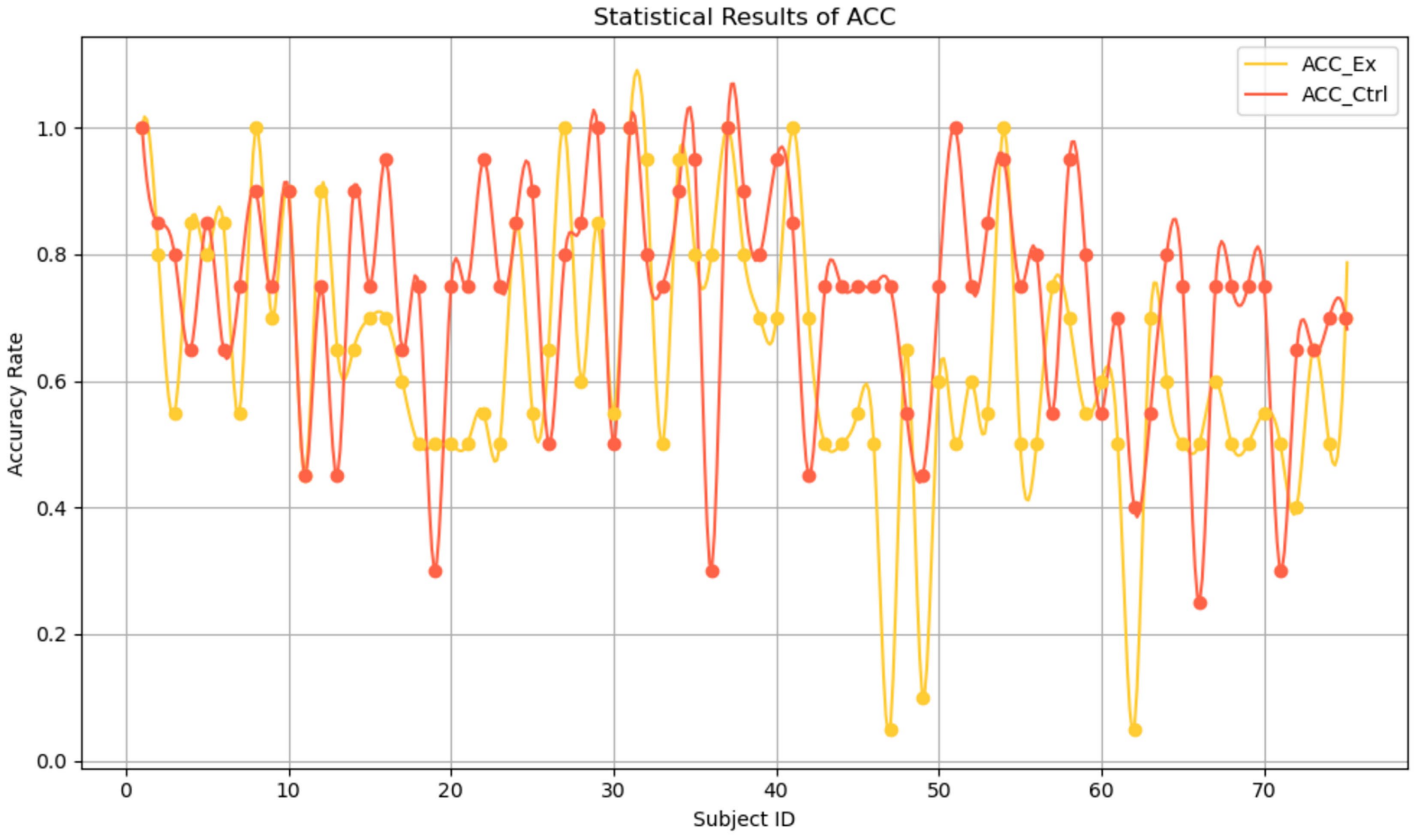
Accuracy rates across participants for experimental and control irony conditions. Each line represents a participant’s accuracy rate in the experimental irony condition (ACC_Ex, yellow line) and the control irony condition (ACC_Ctrl, red line). The y-axis indicates the proportion of correct responses (0.0–1.0), and the *x*-axis represents individual subject ID (1–75). Only ironic sentences were included in the accuracy analysis; literal baseline sentences were excluded due to lack of inferential demands.

As predefined in Section 2.2, accuracy analysis focused exclusively on ironic utterances (experimental vs. control groups) where pragmatic inference is required. Baseline sentences were excluded because their literal meaning permits only objective true/false judgments, unlike irony which demands speaker-intention inference. Therefore, paired-sample correlation was only computed between experimental and control conditions, as both involve inferential processing, unlike the baseline group whose literal comprehension lacks such variability and is not meaningful for inference-based correlation analysis. The accuracy rates of the 75 participants in the experimental and control groups are shown in Figure 4, which preliminarily suggests that the experimental group with stronger reverse adjacency relations had slightly lower accuracy in intention comprehension compared to the control group.

#### 3.3.1 Descriptive statistical analysis

Table 6 displays the mean accuracy rates and standard deviations under different sentence-type conditions. The average accuracy of the experimental group (ACC_Ex) was 0.6413 with a standard deviation of 0.20540; and that of the control group (ACC_Ctrl) was 0.7360 with a standard deviation of 0.18132. This indicates that ironic sentences with clear reverse adjacency relations were more difficult to comprehend, resulting in lower accuracy in the experimental group compared to the control group.

**Table 6:** Descriptive statistics of accuracy rates for experimental and control irony conditions.

Pair	ACC type	Mean	Std	Standard error of the mean	Number of samples
1	ACC_Ex	0.6413	0.2054	0.0237	75
	ACC_Ctrl	0.7360	0.1813	0.0209	75

ACC_Ex = Accuracy rate for experimental irony sentences (with reverse adjacency relations); ACC_Ctrl = Accuracy rate for control irony sentences (without reverse adjacency relations). Std = Standard deviation. Standard Error of the Mean reflects variability across participants (N = 75 for both conditions). Accuracy rates are proportions ranging from 0 to 1.

#### 3.3.2 Paired-sample correlation

According to the analysis results of the correlation between paired samples in Table 7, the correlation coefficient between the experimental group and the control group is 0.426, indicating a moderate positive correlation. Additionally, the *p*-value of the correlation test is less than 0.001, indicating that this correlation is statistically significant. Therefore, it can be confirmed that there is a significant positive correlation between the experimental group and the control group.

**Table 7:** Paired-sample correlation between accuracy rates in experimental and control conditions.

Pair	Condition pair	Number of samples	Correlation	Significance
1	ACC_Ex and ACC_Ctrl	75	0.426	1.4*10^(−5)

ACC_Ex = Accuracy rate in the experimental irony condition (with reverse adjacency relations); ACC_Ctrl = Accuracy rate in the control irony condition (without reverse adjacency relations); Correlation = Pearson product–moment correlation coefficient; Significance = Two-tailed *p*-value.

#### 3.3.3 Paired-sample *t*-tests

The paired-sample *t*-tests results from SPSS in Table 8 reveal a significant difference in accuracy between the experimental and control groups. The mean accuracy rate for the experimental group was 0.6413, while for the control group it was 0.7360, with a difference of −0.09467 and a standard deviation of 0.20821. The *t*-value was −3.938 with 74 degrees of freedom, and the *p*-value was 1.84 × 10^−4^, far below 0.05, indicating that the difference in accuracy between the two groups was statistically significant. The 95% confidence interval was [−0.142, −0.046], which does not include zero, further confirming the significance of the accuracy difference. This suggests that ironic sentences with clear reverse adjacency relations are more complex to comprehend, increasing the difficulty for participants and resulting in lower accuracy in the experimental group. These findings provide empirical support for the role of reverse adjacency relations in irony comprehension, indicating that such relations may play a significant cognitive processing role in the comprehension process.

**Table 8:** Paired-sample t-tests comparing accuracy rates between experimental and control conditions.

Comparison pair	Mean	Std	Standard error of the mean	95% interval lower bound	95% interval upper bound	*t*-value	df	*p*-value
ACC_Ex – ACC_Ctrl	−0.095	0.209	0.024	−0.142	−0.046	−3.938	74	1.84*10^(−4)

ACC_Ex = Accuracy rate in the experimental irony condition (with reverse adjacency relations); ACC_Ctrl = Accuracy rate in the control irony condition (without reverse adjacency relations); Std = Standard deviation; *t*-value = Test statistic for paired-sample *t*-tests; df = Degrees of freedom; *p*-value = Two-tailed significance level; Confidence interval reflects 95% CI for the mean difference.

#### 3.3.4 Paired-sample effect size

The effect size analysis in Table 9 shows that Cohen’s d was 0.455 (absolute value), which falls into the medium effect size category (0.2 for small, 0.5 for medium, and 0.8 for large effects). The adjusted Hedges’ *g* was 0.452, similar to Cohen’s d. This indicates that while the difference in accuracy between the experimental and control groups was significant, its magnitude was moderate. The 95% confidence interval was[−0.691, −0.215], demonstrating a high level of confidence in the effect size, and it does not include zero, confirming the reliability of the result. These analyses further validate the role of reverse adjacency relations in irony comprehension, indicating that the difference is statistically meaningful.

**Table 9:** Paired-sample effect size estimates for accuracy difference between experimental and control conditions.

Effect size type	Standardized value	Point estimate	95% confidence interval, lower bound	95% confidence interval, upper bound
Cohen’s *d*	0.20821	−0.455	−0.691	−0.215
Hedges’ *g*	0.20927	−0.452	−0.688	−0.214

Cohen’s *d* and Hedges’*g* represent standardized effect sizes comparing accuracy between experimental (ACC_Ex) and control (ACC_Ctrl) irony conditions. Negative values reflect lower accuracy in the experimental group. 95% CIs that exclude zero indicate significant effects.

### 3.4 Correlation analysis

In order to examine the general relationship between reaction time and accuracy across ironic conditions, we averaged the reaction times of the experimental group (RT_Ex) and control group (RT_Ctrl) to compute RT_Avg, and similarly averaged their accuracies to compute ACC_Avg. A Pearson correlation analysis was conducted to assess the linear association between these two aggregated measures.

This correlation analysis was limited to the experimental and control irony conditions, as both required participants to infer the speaker’s ironic intent when selecting evaluations (i.e., distinguishing literal meaning from intended criticism/praise). Although the explicit task was binary evaluation selection ([positive]/[negative]), accurate responses depended on pragmatic inference of irony, creating shared cognitive demands across these conditions.

In contrast, the baseline condition involved literal statements where evaluations matched surface meaning (e.g., a positive statement required [positive] selection), demanding no pragmatic inference. Consequently, its fundamentally different processing mechanisms precluded inclusion in irony-specific correlation analyses.

**Figure 5:**
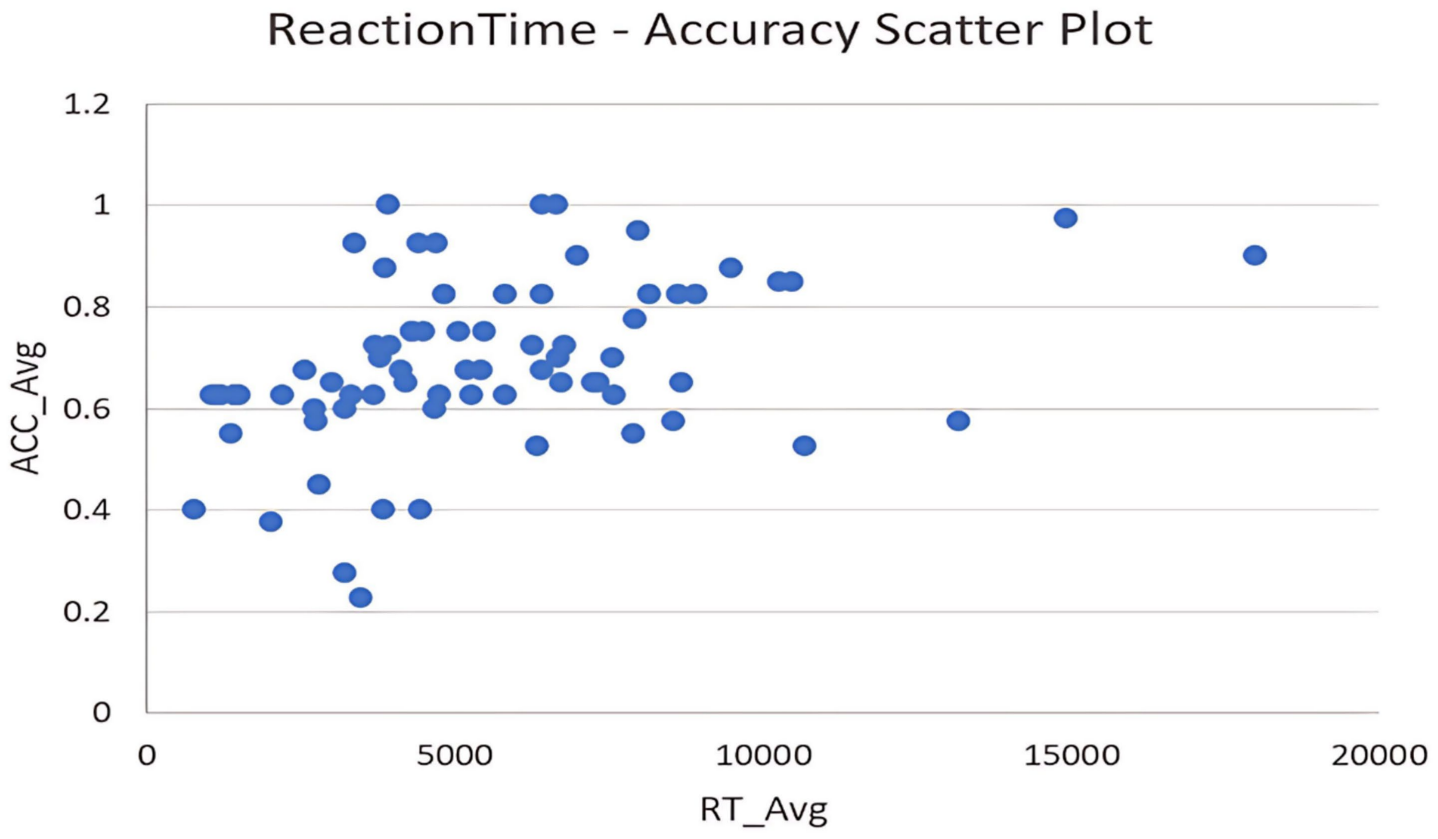
Scatter plot of reaction time (RT_Avg) and accuracy (ACC_Avg) across ironic conditions. Each blue dot represents an individual participant’s averaged reaction time (RT_Avg) and accuracy (ACC_Avg) across experimental and control irony conditions. The observed moderate positive trend supports a speed–accuracy tradeoff: participants with shorter RTs tended to have higher accuracy in irony comprehension.

The results of the correlation analysis are presented in Table 10 and Figure 5.

**Table 10:** Pearson correlation matrix for RT_Avg and ACC_Avg across ironic conditions.

Variables	RT_Avg	ACC_Avg
RT_Avg	1 (0.000***)	0.4 (0.000***)
ACC_Avg	0.4 (0.000***)	1 (0.000***)

RT_Avg = average reaction time across experimental and control irony conditions; ACC_Avg = average accuracy across the same conditions. Values in parentheses indicate two-tailed *p*-values. ***, ** and * represent the significance levels of 1, 5, and 10%, respectively.

The Pearson correlation coefficient between RT_Avg and ACC_Avg was found to be *r* = 0.40, significant at the 1% level (*p* < 0.01), indicating a moderate positive association between reaction time and accuracy. This suggests that participants who responded more slowly tended to perform more accurately. The coefficient of determination, *R*^2^ = 0.16, implies that approximately 16% of the variance in accuracy can be linearly explained by reaction time, with the remaining variation likely attributable to other factors not captured in this analysis.

As illustrated in the scatter plot (Figure 5), the overall pattern supports the presence of a speed-accuracy tradeoff in irony comprehension: participants who took more time tended to achieve higher accuracy.

Only one correlation was performed in this section; thus, no correction for multiple comparisons was required. While the moderate *R*^2^ value suggests that other factors beyond processing speed may contribute to irony comprehension accuracy, identifying such variables remains an open direction for future research.

## 4 Discussion

The present study investigated the cognitive mechanisms underlying irony comprehension in Mandarin Chinese, with a specific focus on the role of reverse adjacent relations (Huang, 2008b). By employing a self-paced reading task and judgment task, the findings revealed that ironic sentences with explicit reverse adjacent relations elicited significantly longer reaction times (RT) and lower accuracy rates compared to control ironic sentences and non-ironic baseline sentences.

Different types of sentences elicited distinct cognitive processing durations, supporting the hypothesis that RTs of comprehension of irony differ significantly between sentences with reverse adjacent relations and those without such features. Thus, reverse adjacent relations substantially impact irony comprehension, requiring greater cognitive resources to process ironic sentences with such structural features.

These results align with Huang’s (2008a, 2008b) theoretical proposition that irony comprehension relies on the cognitive recognition and manipulation of gradient reverse adjacency within conceptual hierarchies. This discussion contextualizes these findings within the broader framework of cognitive experimental pragmatics, evaluates their implications for existing theories of irony, and explores methodological considerations and future research directions.

### 4.1 Reverse adjacency relations and existing models of irony

The study’s findings challenge traditional models of irony processing, such as Grice’s (1975) Standard Pragmatic Model (SPM), which posits that irony comprehension requires a sequential analysis of literal meaning followed by pragmatic inference. The prolonged RT for experimental ironic sentences contradicts SPM’s assumption of mandatory literal-first processing, instead supporting Gibbs (1994) Direct Access Model, which emphasizes immediate contextual integration. However, even Gibbs’ framework does not fully account for the observed accuracy decline in sentences with reverse adjacency relations. This suggests that Huang’s (2008b) theory introduces a novel layer to irony comprehension by highlighting the cognitive cost of resolving graded reverse adjacency relations—a specific form of relational knowledge requiring stable hierarchical gradability between lexical items [e.g., affective polarity scales like “讨厌 (annoying)” → “喜欢 (liking)”]. Crucially, our experimental stimuli were systematically coded to operationalize this construct: 20 adjective pairs with validated gradient oppositions [e.g., “快/慢 (fast/slow),” “聪明/愚蠢 (smart/stupid)”] were selected from Huang’s (2008b) corpus, ensuring relational categories relied on adjacency hierarchies rather than simple antonymy or pragmatic negation. This design explicitly distinguishes Huang’s framework from generic semantic reversal mechanisms.

Similarly, Sperber and Wilson’s (1986) echoic mention theory, which frames irony as a reflection of the speaker’s evaluative stance, struggles to explain how reverse adjacency relations modulate comprehension difficulty. For instance, in the experimental stimulus “你可真是让人讨厌啊” (You are such an annoying person), the irony arises not from echoing a prior proposition but from reversing adjacent semantic relations [e.g., “讨厌(annoying)” vs. “喜欢(liking)”]. This aligns with Xu’s (2007) model-based pragmatic reasoning, which posits that conventional relational knowledge structures guide inferential processes. The findings thus extend echoic theory by emphasizing relational hierarchies as a cognitive foundation for irony.

The Graded Salience Hypothesis (GSH; Giora, 1997), which prioritizes salient meanings during comprehension, also faces limitations in explaining the role of reverse adjacency. While GSH predicts that conventional irony is processed efficiently due to salience, the experimental group’s lower accuracy appears inconsistent with this expectation. This suggests that reverse adjacency may introduce additional cognitive demands not fully accounted for by salience alone, though further research controlling for lexical variables (e.g., frequency, familiarity) is needed to clarify this interaction.

The observed RT-accuracy correlation (Pearson’s *r* = 0.4, *p* < 0.001) reflects a classic speed-accuracy tradeoff (Wickelgren, 1977), where faster responses correlated with higher accuracy. However, the moderate correlation leaves 84% of variance unexplained, suggesting additional factors influence irony comprehension. For example, individual differences in working memory capacity (Filik et al., 2017) or familiarity with ironic conventions (Kwon et al., 2020) may modulate processing efficiency. Future studies could incorporate these variables to refine Huang’s framework.

### 4.2 Enhancing cross-cultural models of irony

Our findings significantly advance cross-cultural pragmatics by demonstrating that reverse adjacency relations constitute a culture-specific cognitive mechanism central to irony processing in Mandarin Chinese. This challenges strictly universalist accounts of figurative language comprehension (e.g., Gricean maxim violations as sufficient; Grice, 1975) and strongly supports Colston’s (2000) seminal argument that irony interpretation cannot be reduced to universal pragmatic principles alone. Instead, it is fundamentally constrained by culturally shared communicative norms and locally shaped pragmatic expectations. The prominence of structured relational hierarchies in Mandarin irony underscores that cognitive processes for figurative language are culturally scaffolded, necessitating culturally grounded frameworks for irony understanding (e.g., extending work by Dynel, 2013; Kapogianni, 2016).

Theoretically, our results indicate that relational reversals operate within culturally defined boundaries, challenging claims of cognitive universality. This provides empirical evidence against strong universalist models, highlighting the critical role of cultural schemas in irony comprehension. Specifically, the cognitive mechanisms for irony (e.g., relational reversal) may be modulated by cultural-linguistic systems rather than being fixed universals. These findings align with and extend the Graded Salience Hypothesis (Giora, 2003) and Attardo’s (2000) General Theory of Verbal Humor (GTVH), emphasizing that the “opposition” parameter in irony relies on culturally salient adjacency relations.

From a cross-linguistic perspective, Mandarin’s paratactic syntax and reliance on semantic juxtaposition (versus hypotactic languages like English; Li and Thompson, 1981) may amplify the salience of reverse adjacency patterns in irony. Future research should investigate whether linguistic typology (e.g., parataxis) or cultural frameworks (e.g., high-context communication; Hall, 1976) primarily modulate this mechanism, underscoring the need for typologically informed models of figurative language processing.

Practically, these insights offer concrete applications:

1. Language Pedagogy: Explicit instruction in relational contrasts within cultural contexts (e.g., contrasting expected versus reversed adjacency hierarchies) could enhance irony comprehension for L2 Mandarin learners, reducing cognitive load and mirroring successes in EFL pedagogy (Prichard et al., 2024).
2. Natural Language Processing: Integrating structured relational knowledge frameworks (Xu, 2007) —particularly culture-specific adjacency expectations— could address LLMs’ current limitations in irony detection (Liu and Qi, 2024) by enhancing pragmatic reasoning and contextual modeling.

### 4.3 Limitations and future directions

Despite careful control over certain aspects of stimulus design—such as sentence and context length (character count)—the present study did not formally control for several potentially influential psycholinguistic variables, including character stroke count, lexical frequency, and tone distribution. These factors may subtly affect reading fluency and reaction time in Chinese text processing, thereby introducing unintended variability. Future studies are encouraged to incorporate these linguistic variables into the construction and validation of materials to better isolate the cognitive effects of pragmatic features such as reverse adjacency. Additionally, implementing a more fine-grained gradient manipulation of irony (e.g., multiple levels of reversibility rather than categorical groups) may help more precisely test the theoretical continuum proposed in prior work. Such methodological refinements will further advance the empirical study of Mandarin irony comprehension within cognitively grounded frameworks.

## 5 Conclusion

This study fundamentally advances our understanding of irony cognition by establishing reverse adjacency relations as a culture-specific cognitive mechanism pivotal to Mandarin irony processing. Theoretically, our findings challenge universalist frameworks (e.g., Gricean maxim violations as sufficient explanations) and necessitate culturally attuned models that integrate relational hierarchies and socio-pragmatic norms—aligning with Colston’s (2000) contention that irony comprehension is irreducibly localized. Crucially, we demonstrate that cognitive operations like relational reversal are modulated by cultural-linguistic systems, not fixed universals, thereby redefining the “opposition” parameter in irony theories (e.g., Attardo’s GTVH; Giora’s Salience Hypothesis). Practically, this research pioneers dual pathways for applied innovation: in L2 pedagogy, it transforms irony instruction from intuitive exposure to rule-based relational contrast training, reducing cognitive load through culturally scaffolded hierarchies (e.g., “expected vs. Reversed” adjacency drills); in AI advancement, it enables NLP systems to overcome irony detection limitations by injecting culture-specific relational knowledge frameworks (Xu, 2007), enhancing pragmatic reasoning in LLMs. Methodologically, we bridge disciplines through synergistic integration—validating Xu’s (2007) model-based reasoning with behavioral experiments while uniting linguistics, psychology, and cognitive science to decode the triadic interplay of language structure (e.g., Mandarin parataxis), cultural norms (e.g., hierarchical adjacency), and cognitive mechanics. Looking ahead, future research must dissect how syntactic structures (e.g., hypotaxis vs. parataxis) and cultural schemas (e.g., high−/low-context) co-modulate irony cognition across typologically diverse languages. By exposing culture as the scaffold of figurative thinking, this work not only reorients irony theory beyond Western-centric assumptions but establishes a paradigm for computationally modeling the dynamic nexus of language, cognition, and culture.

## Data Availability

The raw data supporting the conclusions of this article will be made available by the authors, without undue reservation.
